# A direct comparison of the effects and mechanisms between species richness and genotype richness in a dominant species on multiple ecosystem functions

**DOI:** 10.1002/ece3.8125

**Published:** 2021-09-16

**Authors:** Man Jiang, Xue Yang, Tao Wang, Yujuan Xu, Ke Dong, Luoyang He, Yulin Liu, Jinlong Wang, Nianxi Zhao, Yubao Gao

**Affiliations:** ^1^ Department of Plant Biology and Ecology College of Life Science Nankai University Tianjin China; ^2^ College of Agronomy & Resources and Environment Tianjin Agricultural University Tianjin China

**Keywords:** biodiversity and ecosystem functioning, community‐weighted mean trait values, functional dispersion, interspecific richness, intraspecific richness

## Abstract

Both species (interspecific) richness and genotype (intraspecific) richness of dominant species have significant effects on ecosystem functioning directly or indirectly by regulating plant community functional structure. However, the similarities and differences of the effects between inter‐ and intraspecific levels are poorly understood. In this study, we selected the main species in the semi‐arid Eurasian typical steppe as study objects and simultaneously carried out a species richness experiment and a genotype richness experiment of *Stipa grandis* which is one of the dominant species in this region. We investigated how plants at each of the two richness levels affected multiple ecosystem functions (biomass, soil C, N and P cycles) directly and indirectly by regulating community functional structure, including community‐weighted mean trait values (CWM) and functional dispersion (FDis). Both species richness and genotype richness showed significant direct effects on soil P cycle, and FDis significantly mediated the responses of aboveground biomass and soil N cycle to the changes of species richness and the response of belowground biomass to the changes of genotype richness in *S. grandis*. CWM showed significant effects on biomass in the species richness experiment and soil nutrient cycles in the genotype richness experiment, independently of the levels of plant richness. These findings provide experimental insights of intraspecific richness effects into the relationships between biodiversity and ecosystem functioning, and highlight the importance of conserving the intraspecific diversity of dominant species in the semi‐arid steppe regions.

## Graphical abstract

Both species and genotype richness showed significant direct effects on soil P cycle; FDis regulated the responses of aboveground biomass and soil N cycle to the changes of species richness; FDis regulated the response of belowground biomass but not soil functions to the changes of genotype richness.

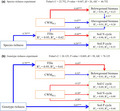

## 1 INTRODUCTION

Due to the rapid loss of biodiversity caused by global changes and human disturbances, more and more studies have focused on the relationships between biodiversity and ecosystem functioning (BEF) (Benayas et al., 2009; Maestre et al., 2012). Over years, most studies have confirmed that species richness has significant positive direct effects on a single ecosystem function such as biomass production (van der Plas, 2019). Meanwhile, there is increasing evidence that genotype richness plays an important role in affecting a single ecosystem function (Schweitzer et al., 2011; Souza et al., 2016). Therefore, exploring the processes and mechanisms by which plant diversity at different levels (inter‐ vs. intraspecific) affects ecosystem functioning and examining the similarities and differences of BEF between these two levels are essential for predicting the effects of biodiversity loss on ecosystem functioning (Raffard et al., 2019).

In recent years, there has been an increasing number of studies on the ability of ecosystems to simultaneously provide multiple ecosystem functions and services (multifunctionality). Biomass production, which represents the ability and efficiency of plants in a community to utilize natural resources, is the most widely used ecosystem function in BEF researches (van der Plas, 2019). Besides, soil nutrient cycles, which reflect the ecosystem resource utilization processes (some basic support and regulation of ecosystem services), are very sensitive to the changes in community composition such as plant richness; therefore, soil nutrient cycles have attracted more and more attention in BEF researches (Maestre et al., 2009). When multiple ecosystem functions are considered in one study, relationships among different ecosystem functions need to be understood. Although diversity could increase ecosystem functions simultaneously, some functions may inherently trade off (Lefcheck, 2015; Wu et al., 2019); soil organic carbon content and reserves (C cycle) and biomass production are one such example (Chen et al., 2019; Prommer et al., 2020). Moreover, environmental conditions or dominant species may cause ecosystem functions to trade off with one another (Gamfeldt et al., 2013).

The effects of species richness on ecosystem functions are usually explained by the mass ratio hypothesis and niche complementarity hypothesis which are driven by community functional structure, including community‐weighted mean trait values (CWM) and functional dispersion (FDis) (Mouillot et al., 2011; Valencia et al., 2018). The mass ratio hypothesis proposes that the traits of dominant species mediate the responses of ecosystem functions to the changes of plant communities largely (Grime, 1998; Valencia et al., 2015), and CWM could reflect the traits of dominant species because it is calculated based on species trait values and species relative abundance (Díaz et al., 2007). The niche complementarity hypothesis posits that the positive effect of plant diversity on ecosystem functions is primarily mediated by the differences in resource utilization among species which is highly associated with FDis (Díaz et al., 2007; Giling et al., 2019; Tilman et al., 1997). To our knowledge, only a few studies have paid attention to how community functional structure mediates the responses of soil ecosystem functions to the changes of plant richness. In addition, there are significant differences in community functional structure between communities of diverse species and those of a single species with diverse genotypes because of the lower variance of functional traits in a single‐species communities (He et al., 2018). However, few studies have focused on the differences of mechanisms by which diversity at different levels (inter‐ vs. intraspecific) drives the BEF. Therefore, any efforts to explore how and the mechanism by which community functional structure mediates the responses of ecosystem functions to the changes of richness at intra‐ and interspecific levels are helpful for ecologists to fully understand the BEF relationships, especially in the degrading regions.

The semi‐arid temperate steppe of northern China is an important part of the Eurasian Steppe. However, due to the global climate changes and intensive human disturbances such as overgrazing, the inter‐ and intraspecific diversity in this region are being lost (Wang et al., 2000). *Stipa grandis*, one of the important dominant species in this region, has been found the decrease of genotype richness in the degraded community (Chen & Wang, 2000). Recent studies have shown that species richness shows higher net diversity effects on biomass production and litter decomposition than genotype richness in *S. grandis* although there are similar trends of BEF at both levels (Yang, Qu, et al., 2019; Yang, Wang, et al., 2019), and supposed that community functional structure plays an important role in regulating the effects of diversity at both levels (Yang, Wang, et al., 2019). In order to deeply understand the ecological consequences of biodiversity loss in the semi‐arid temperate steppe of northern China, in this study, we further explored how plant diversity at different levels affects multiple ecosystem functions (soil C, N, and P cycle as well as biomass production) and the potential mechanisms accordingly mediated by community functional structure based on two independent richness microcosm experiments by modulating plant species richness and genotype richness in *S. grandis* (See Yang, Wang, et al., 2019). Such information would strengthen the significance of dominant species on ecosystem functioning, improve our ability to interpret the effect of biodiversity loss on ecosystem functioning, and, in turn, lead to better management and conservation of ecosystems. Specifically, we proposed two hypotheses. First, there are some similar effects of richness on ecosystem functioning at both levels. Second, the niche complementarity hypothesis dominates the BEF relationships either in the species richness experiment or genotype richness experiment of the dominant species *S. grandis*.

## 2 MATERIALS AND METHODS

### 2.1 Plant and soil collection

In early May of 2015, during the growing season, soil in 0–20 cm layer and 13 plant species which represent the basic composition of the semi‐arid temperate steppe of northern China, were collected in a typical steppe (116°40′E, 43°32′N). The collected soil was homogenized after removing large stones and plant roots and then used in the following experiments. The soil contained 2.2% clay, 17.6% silt, and 80.2% sand with pH 7.5 and was classified as Calcis‐orthic Aridisol according to US soil taxonomy classification. The collected plants were transplanted and acclimated in nutritional soil for about 2 months in the open experimental field at Nankai University (117°17’E, 39°10’N) before being used for the following experiments.

### 2.2 Experimental design

**FIGURE 1:**
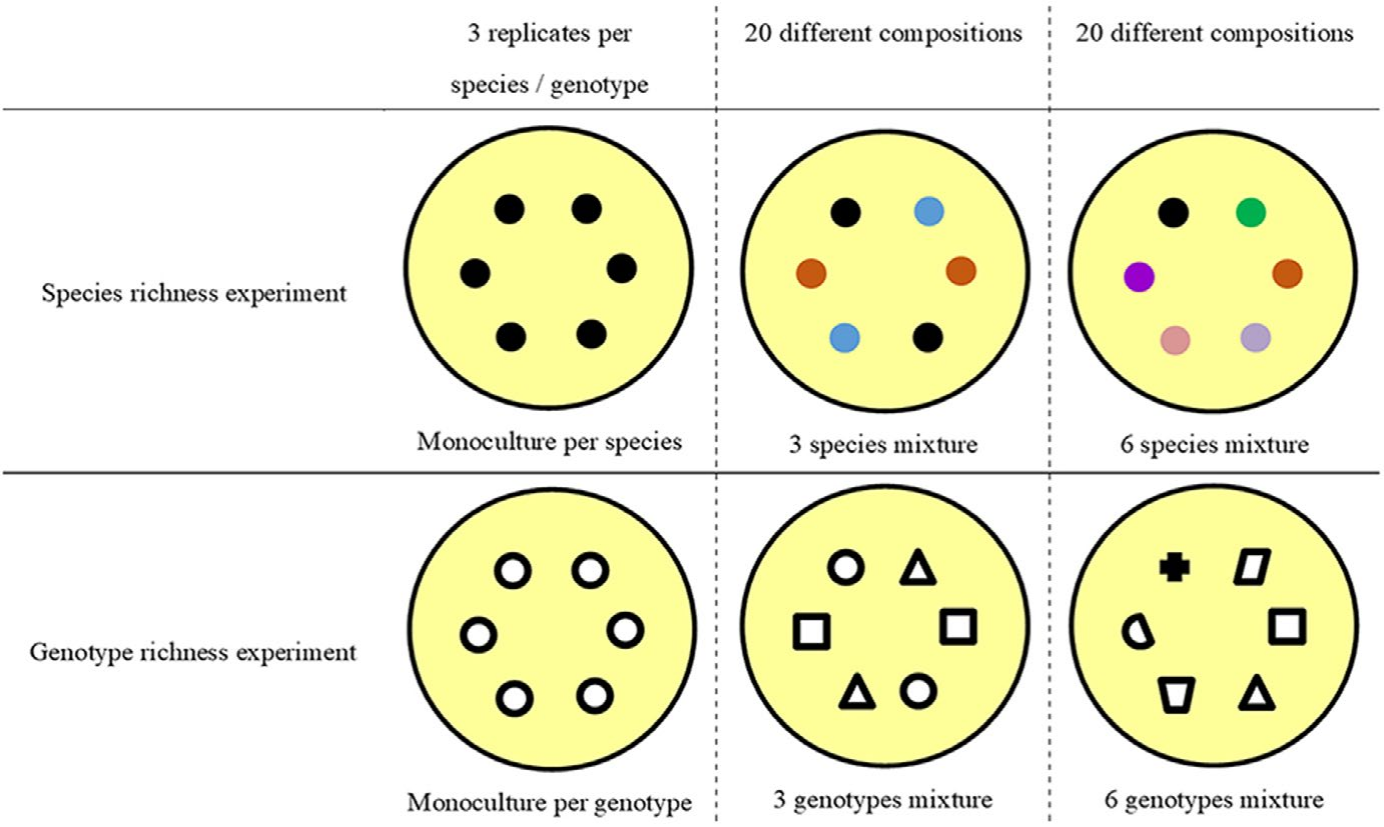
Diagram of experimental design. Each pot (yellow circle with black frame) contained 6 individuals which were randomized and planted equidistant from one another. The number of colors in the species richness experiment represents species richness and the number of shapes in the genotype richness experiment represents genotype richness in *Stipa grandis*

Two independent richness experiments were set up simultaneously (Figure 1). One experiment is the species richness experiment, with one monoculture (1 species) and two mixtures (3 species and 6 species) treatments, and individuals from 12 species which were collected randomly in the sample plot, so that their genetic identities are unknown. The 12 species included six perennial grasses (*Achnatherum sibiricum*, *Agropyron Cristatum*, *Cleistogenes squarrosa*, *Koeleria cristata*, *Leymus chinensis*, *Poa pratensis*), four perennial forbs (*Allium senescens*, *Potentilla bifurca*, *Potentilla acaulis*, *Serratula centauroides*), one sedge (*Carex korshinskyi*), and one semi‐shrub (*Artemisia frigida*). Another experiment is the genotype richness experiment, with one monoculture (1 genotype) and two mixtures (3 genotypes and 6 genotypes), and individuals from 12 genotypes of *S. grandis* were used. Each genotype used in this experiment was determined to be unique using amplified fragment length polymorphisms (AFLPs) (Yang, Wang, et al., 2019).

In early July of 2015, individuals from the same species were treated to similar sizes in plant height and root length (Table S1 in Appendix S1) and transplanted into plastic pots (depth 19 cm, internal diameter 15 cm) according to the experimental design in Figure 1. Six individuals were equally spaced and randomly assigned. Each monoculture treatment was replicated three times per species (or genotype), and each mixture treatment was replicated 20 times with 20 different compositions. A total of 152 microcosms, with 76 microcosms [3 replications × 12 monocultures + 20 different compositions × 2 mixtures] for each independent richness experiment. Each species (or genotype of *S. grandis*) assumed the same likelihood in a certain pot within each treatment, and individuals in mixtures were randomly selected from species and genotype pools mentioned above. Only a few individuals died within the first 2 weeks after transplantation, and they were replaced by healthy individuals of similar sizes.

The experiments were conducted in a experimental field at Nankai University with a 5‐meter‐height rain‐proof shed which allows 95% natural light to penetrate but does not change the ambient temperature and wind. All the pots were placed randomly, and they were changed every 2 weeks to avoid position effect. During the experiment, shade and water stress were avoided and weeds were removed regularly once a week. The experiment lasted for 20 weeks and ended at the end of November of 2015.

### 2.3 Measurement of plant functional traits

From the end of October to the end of November 2015, plant functional traits that are related to plant growth rate, competitiveness, and resource utilization, including plant height, plant width, specific leaf area (SLA), leaf dry matter content (LDMC), root volume, and leaf C, N, P content, were determined for each species or each genotype of *S. grandis*, by standard methods (Cornelissen et al., 2003; Schöb et al., 2015). Plant height is the vertical distance from the upper end of the photosynthetic tissue of the plant to the soil surface. Plant width is the widest horizontal distance between the two ends of the photosynthetic tissue. SLA is the ratio of leaf area to leaf dry weight. LDMC is the ratio of leaf dry weight to leaf saturated water mass. Root volume was measured using water displacement. Leaf C and N contents were measured using an elemental analyzer (Elementar, Germany). Leaf P content was measured by molybdenum antimony scandium colorimetric method. The leaves were powdered by MM301 (Retsch, Germany) before measuring leaf C, N, and P contents. Each trait included at least three replicates per species or per genotype of *S. grandis*, and the mean value was used to calculate community functional structure.

### 2.4 Measurement of ecosystem functions

At the end of November 2015, aboveground shoots and belowground roots in each pot were carefully harvested by individuals and then dried at 80℃ for 48 hr to obtain the aboveground and belowground biomass of individual species or genotype of *S. grandis*, respectively. The biomass of individuals in each pot was used to measure the community functional structure. For each microcosm, the aboveground biomass (g·pot^−1^) was the sum of the leaf dry mass used to measure functional traits plus the aboveground dry mass of all individuals; the belowground biomass (g·pot^−1^) was the sum of belowground dry mass of all individuals. Some tiny roots (lower than 1% of the total belowground) that dropped from the plant during the harvest were not included in the belowground biomass because it was very difficult to separate and identify them by species. The rhizosphere soil was collected for each pot and used for the measurement of soil C, N and P cycles which have been proved good proxies for ecosystem functions (Maestre et al., 2012). Each soil function included 2–6 variables that were measured using the methods listed in Table 1. The soil samples were sieved by 0.125 mm mesh for the measurement of soil total C and N contents and 2 mm mesh for the measurement of soil available P content, respectively.

**TABLE 1:** Measurement methods for soil C, N, and P cycles

Soil ecosystem function	Variable	Method of measurement
Soil C cycle	Soil total carbon content	Elemental analyzer (Vario MAX C/N‐Macro)
	Soil organic carbon content	Low‐temperature external‐heat potassium dichromate oxidation‐photo‐colorimetric method (Anderson & Ingram, 1994)
	Soil β‐glucosidase activity	Nitrophenol colorimetry method (Suzhou Keming Company kit)
Soil N cycle	Soil total nitrogen content	Elemental analyzer (Vario MAX C/N‐Macro)
	Soil ammonium content (${\text{NH}}_{4}^{+}$‐N)	Indophenol blue colorimetry method (Lu, 2000)
	Soil nitrate content (${\text{NO}}_{3}^{‐}$‐N)	Double wavelength colorimetry (Wang & Tang, 2016)
	Soil nitrification rate	The ion exchange resin bag method (Mo et al., 2001)
	Soil mineralization rate	The ion exchange resin bag method (Mo et al., 2001)
	Soil urease activity	Sodium phenol‐sodium hypochlorite colorimetry method (Suzhou Keming Company kit)
Soil P cycle	Soil available phosphate content	Molybdenum antimony scandium colorimetric method (Zhan et al., 2015)
	Soil phosphatase activity	Disodium phenyl phosphate colorimetry method (Suzhou Keming Company kit)

### 2.5 Data analyses

#### 2.5.1 Community functional structure

All data used in this part met the Shapiro–Wilk test of normality and the Levene's test of homogeneity of variance after the leaf P content data were transformed by COS [Cos (leaf P content) returns the cosine of radians] (SPSS Inc., version 20.0).

The community functional structure, including CWM and FDis, was quantified by two complementary matrices using package “*FD*” in R (R 4.0.3; R Core Team, 2020). The FDis, a multi‐trait index, was calculated based on the values of all functional traits measured in this study. The CWM is a single‐trait index. Before we calculated CWM, we selected two functional axes of traits as functional markers by principal component analysis (PCA) for the assessment of the CWM (R 4.0.3; R Core Team, 2020) (Butterfield & Suding, 2013). First, correlation coefficients between pairwise plant functional traits were calculated by Spearman correlation analysis (SPSS Inc., version 20.0) to judge redundancy among these traits (if the correlation coefficient is higher than 0.7) (Dormann et al., 2013). Then, plant height in the species richness experiment and leaf N content in the genotype richness experiment were omitted according to the correlation coefficients (Table S2 in Appendix S1) before we performed PCA (Valencia et al., 2018). Second, for species richness experiment, the first two PCs explained 64.2% of the total variance in the data. The PC1, which was mainly contributed by SLA, was classified as light competition axis; and the PC2 mainly that was contributed by leaf C content was classified as conservative resource utilization axis (Wilson et al., 2002) (Table 2). For genotype richness experiment, the first two PCs explained 59.8% of the total variance in the data. Both the PC1 mainly contributed by LDMC and root volume and the PC2 contributed by leaf C content were considered as conservative resource utilization axis (Westoby et al., 2002) (Table 2). Third, the PC scores of the first two axis were obtained and used for quantifying CWM that were marked CWM_PC1_ and CWM_PC2_, respectively.

**TABLE 2:** Eigenvectors of the trait variables used in the principal component analysis

Plan functional traits	Species richness experiment		Genotype richness experiment	
	First component (35.27%)	Second component (28.96%)	First component (36.08%)	Second component (23.69%)
Plant height	–	–	0.05	−0.21
Plant width	0.79	0.12	−0.48	0.03
Specific leaf area	**0.93**	0.17	0.69	0.53
Leaf dry matter content	0.42	0.51	**0.95**	0.05
Root volume	0.66	−0.38	**0.82**	0.16
Leaf C content	0.29	**0.88**	−0.44	**0.83**
Leaf N content	−0.23	0.49	–	–
Leaf P content	−0.48	0.75	−0.2	0.79

Note

The eigenvectors higher than 0.8 are in bold style.

#### 2.5.2 Soil functions

First, to judge redundancy (if the correlation coefficient is higher than 0.7) among these soil variables, correlation coefficients between pairwise soil variables were evaluated by Spearman correlation analysis (SPSS Inc., version 20.0). No correlation coefficient was higher than 0.7 (Table S3 in Appendix S1); thus, no variable was omitted before further analysis. Second, the C, N, and P cycles functions were calculated using the average method based on the soil variables accordingly using package “*multifunc*” in R (R 4.0.3; R Core Team, 2020).

### 2.6 Statistical analyses

One‐way analysis of variance (ANOVA) was used to evaluate the effects of plant richness (species or genotype richness) on ecosystem functions (aboveground biomass, belowground biomass, C, N, P cycles) (SPSS Inc., version 20.0). Moreover, the magnitude of effects (*ω* ^2^) of individual factor was calculated by dividing each variance component to the total variance (Graham & Edwards, 2001).

To test the direct and indirect causal relationships between predictors and each ecosystem function (aboveground biomass, belowground biomass, and C, N, P cycles), a confirmatory path analysis was constructed for each experiment using Shipley's test of *d*‐separation (Lefcheck, 2015) using package “*piecewiseSEM*” in R (R 4.0.3; R Core Team, 2020), with species/genotype richness, and community functional structure (CWM_PC1_, CWM_PC2_ and FDis) as fixed factors, plant combination as a random factor. Meanwhile, the relationships of pairwise ecosystem functions were estimated. A *priori* model is established based on theoretical knowledge (Figure S1 in Appendix S1). We simplified the models by removing non‐significant pathways based on regression weights. If several models were accepted, we selected the model with the smallest AIC as the final model. Finally, standardized path coefficients were used to measure the direct, indirect effects of the predictors.

## 3 RESULTS

### 3.1 Effect of plant richness at intra‐ or interspecific level on individual ecosystem function

Both species richness and genotype richness in *S. grandis* showed significant positive effects on aboveground biomass ($\omega_{\text{species}}^{2}$ = 15.170%; $\omega_{\text{genotype}}^{2}$ = 8.819%) and soil P cycle ($\omega_{\text{species}}^{2}$ = 34.762%; $\omega_{\text{genotype}}^{2}$ = 43.125%), and non‐significant effects on belowground biomass and soil C cycle. Species richness significantly negatively affected soil N cycle (*ω* ^2^ = 19.176%) but genotype richness did not (Table 3).

**TABLE 3:** The effects of species richness or genotype richness in *Stipa grandis* on ecosystem functions and the corresponding direction (↑ or ↓) and magnitude (*ω* ^2^) of effect

Variable	Species richness (*df* = 2)			Genotype richness in *S. grandis* (*df* = 2)		
	*F*	*p*‐value	Magnitude of effects (*ω* ^2^) (%)	*F*	*p*‐value	Magnitude of effects (*ω* ^2^) (%)
Aboveground biomass	6.527	.**002↑**	15.170	3.530	.**034↑**	8.819
Belowground biomass	2.843	.065	7.227	0.740	.480	2.015
Soil C cycle	1.389	.261	6.203	1.629	.208	7.199
Soil N cycle	4.982	.**011↓**	19.176	0.888	.419	4.058
Soil P cycle	11.190	<.001↑	34.762	15.923	<.001↑	43.125

Note

↑ represents the significant positive effect, and ↓ represents the significant negative effect of factors on the ecosystem function variable, respectively.

### 3.2 The direct and indirect effects of plant richness at intra‐ or interspecific level on multiple ecosystem functions

#### 3.2.1 Species richness experiment

**FIGURE 2:**
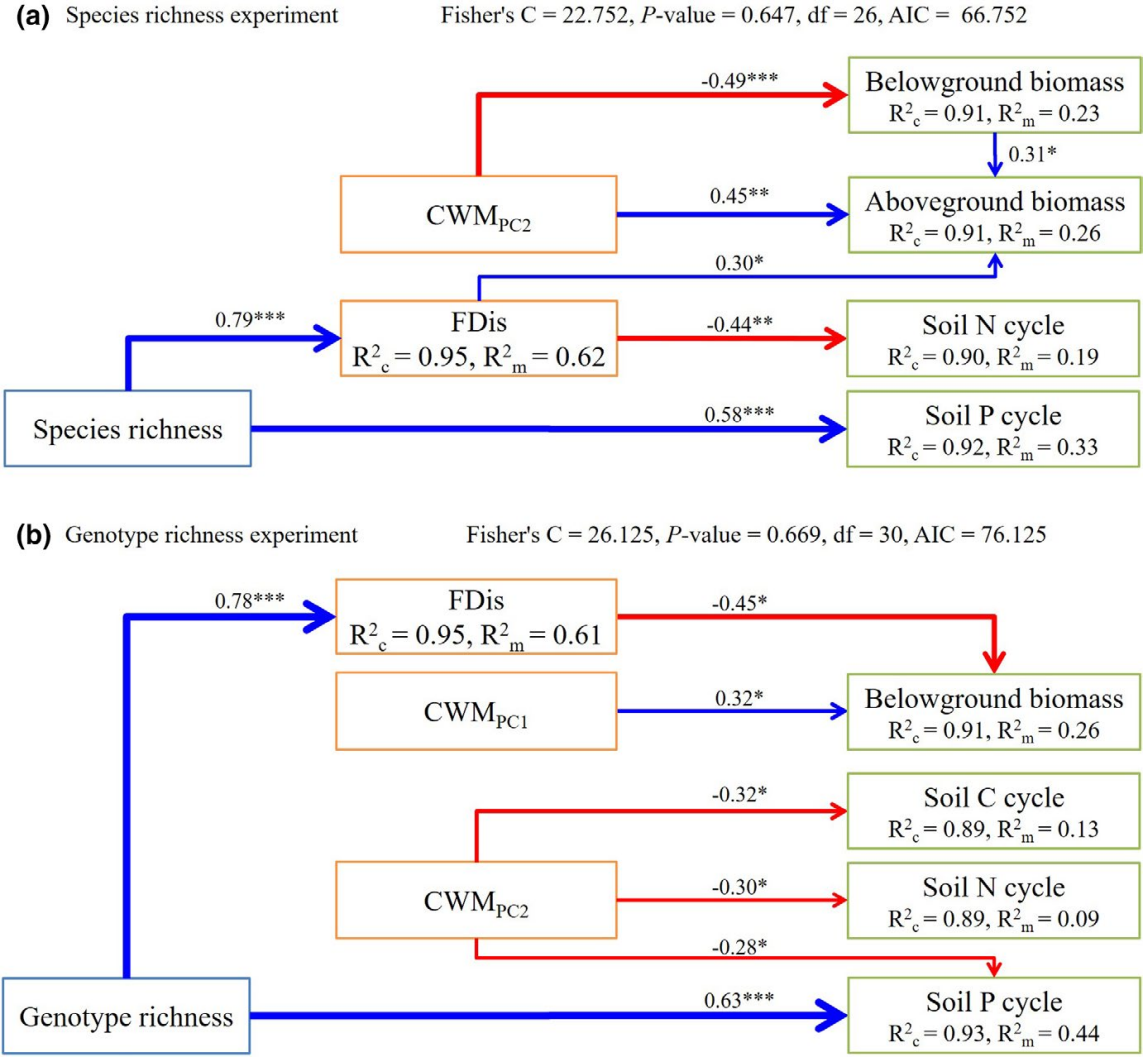
The casual relationships between plant richness (rectangle with blue frame) at the interspecific level (a) and intraspecific level of *Stipa grandis* (b), community functional structure (CWM_PC1(PC2)_, community‐weighted mean the first and second functional marker value by PCA; and FDis, functional dispersion) (rectangle with orange frame); and multiple ecosystem functions (rectangle with green frame). Blue and red arrows represent the significant positive and negative pathways, respectively. *, **, and *** represent the significance level at 0.05, 0.01, and 0.001 levels, respectively. Numbers along the arrows are standardized path coefficients, indicating the effect sizes of the relationships. $R_{c}^{2}$: variance explained by both fixed and random factors; $R_{m}^{2}$: variance explained by fixed factor

The final model explained at least 90% of the total variance in each ecosystem function. Species richness had a significantly direct effect on soil P cycle with the standardized path coefficient being 0.58 but not any of the other ecosystem functions. Species richness showed significant indirect effects on aboveground biomass and soil N cycle by regulating the FDis, with the standardized path coefficient being 0.30 and −0.44, respectively. The CWM_PC2_ showed a positive direct effect on aboveground biomass and a negative direct effect on belowground biomass, while it did not mediate the indirect effect of species richness on biomass (Figure 2a). The belowground biomass was positively associated with aboveground biomass.

#### 3.2.2 Genotype richness experiment

The final model explained at least 89% of the total variance in each ecosystem function. Genotype richness had a significant direct effect on soil P cycle with the standardized path coefficient being 0.63 but not any of the other ecosystem functions. Genotype richness showed a significant indirect effect on belowground biomass by regulating the FDis, with the standardized path coefficient being −0.45. The CWM_PC1_ showed a positive direct on belowground biomass, and the CWM_PC2_ showed negative direct effects on soil C, N, and P cycles. However, both CWM_PC1_ and CWM_PC2_ did not mediate the indirect effect of genotype richness on the ecosystem functions (Figure 2b).

## 4 DISCUSSION

The present study represents an attempt to evaluate how plant richness at different levels affects multiple ecosystem functions directly and indirectly by regulating the community functional structure by two independent richness experiments. These findings provide empirical evidence for the potential mechanisms of BEF at species richness and genotype richness in the dominant species *S. grandis* in the semi‐arid temperature steppe of northern China. By direct comparison, the findings at these two richness levels are similar in some aspects but different in others.

### 4.1 The effects of plant richness at intra‐ or interspecific level on ecosystem functions

Our findings showed that either species richness or genotype richness had significant effects on aboveground biomass and soil P cycle (Table 3), with positive direct effects on soil P cycle (Figure 2), supporting the first hypothesis. Although the similar effects of species richness and genotype richness in dominant species on biomass have been reported by many studies (Cook‐Patton et al., 2011; Schöb et al., 2015), few studies have shown their similar effects on soil nutrient cycles, especially soil P cycle. As one of the important soil nutrient cycles, P cycle has been positively affected by species richness in various ecosystems (Oelmann et al., 2011; Sorkau et al., 2018). In a study of forest and grassland ecosystem in Germany, Sorkau et al. (2018) have shown a significant positive effect of plant species diversity on the P cycle (microbial P concentration). In an experimental grassland near the city of Jena in German, Oelmann et al. (2011) have found a significant positive effect of diversity on P cycle (P utilization). As for the effect of genotype richness, Cong et al. (2020) have shown that the ability of some cash crops to use P depends on their genotypes; however, the significant positive relationship between genotype richness and P cycle has rarely been reported.

The significant positive relationship between plant species richness and the soil P cycle could be explained by the long‐term adaptation to the low P environment in the study region (Šmarda et al., 2013). The plant materials and soil used in this study are from the semi‐arid typical steppe in northern China, and the soil P content in the whole region is 0.35 ± 0.02 mg·g^−1^, which is a low P level (Geng et al., 2011). Therefore, from the point of view of evolution, different species or different genotypes of the same species in this region would show different adaptive strategies for a low P environment. For example, Shi et al. (2011) have found that *L. chinensis* that was used in our study could enhance the uptake of P by arbuscular mycorrhizal fungi and consequently promote self‐growth. According to the insurance hypothesis, plant communities with greater species richness have a higher chance to contain species that are well adapted to a certain environment, such as P deficiency environment. Besides, species with strong soil P mobilization capacity can promote P utilization by neighboring species (Yu et al., 2020). As a result, greater species richness from a lower P environment like in this study ultimately could show a higher P cycle.

Similar to species richness, the genotype richness in *S. grandis* showed a significant positive effect on the soil P cycle. Our recent studies have found the similar ecological effects of species richness and genotype richness in *S. grandis* on biomass production and litter decomposition (Yang, Qu, et al., 2019; Yang et al., 2021), and indicated that greater genotype richness in *S. grandis* could drive facilitative plant‐plant interaction by trait‐dependent complementarity effect (Yang et al., 2021). All these findings have confirmed that the effects of genotype richness in *S. grandis* on ecosystem functioning are as important as species richness in the semi‐arid temperate steppe of northern China. In the present study, *S. grandis* is not included in the species pool of species richness experiment. Therefore, it is hard to judge the effects of the dominant species' genotype richness on multiple ecosystem functions at the interspecific level (Latta et al., 2011).

### 4.2 The effects of FDis on ecosystem functions

It was FDis but not CWM that mediated the indirect effects of richness on ecosystem functions at either inter‐ or intraspecific level (Figure 2). Greater FDis could increase complementary for resources utilization among plants (Díaz et al., 2007; Tilman et al., 1997), which could explain the positive relationship between FDis and aboveground biomass in the species richness experiment. However, there was a negative relationship between FDis and belowground biomass in the genotype richness experiment (Figure 2b) and between FDis and soil N cycle in the species richness experiment, which is out of expectation. Therefore, the present findings support the second hypothesis partly. These findings might result from the low degree of niche differentiation within species and less utilization of resources in marginal niches (Wang et al., 2019). Recently, some studies have shown unimodal relationships but not linear relationships between plant richness and ecosystem functions such as biomass production on the worldwide scale (Fraser et al., 2014; Yang et al., 2021). In a treeless community, the species richness–pH relationship has been found unimodal in the Western Sayan Mountains, southern Siberia (Chytrý et al., 2007). Besides, a study has proposed that a unimodal relationship should be tested between species richness and aboveground plant biomass plus dead plant litter (Fraser et al., 2014).

### 4.3 The effects of CWM on ecosystem functions

The present study shows that the CWM did not mediate the responses of ecosystem functions to the changes of richness at either inter‐ or intraspecific level, although the significant direct effects of CWM on ecosystem functions were observed (Figure 2). In a full factorial mesocosm experiment at the facilities of Rey Juan Carlos University, Valencia et al. (2018) have shown that CWM_SLA_ is not regulated by species richness but can directly affect ecosystem multifunctionality in the presence of climate change when they studied the BEF relationships in typical grasslands in central Spain. The above findings indicate that the mass ratio hypothesis dominated the responses of ecosystem functions via community functional structure, independently of the levels of plant richness. Moreover, in both richness experiments, the PC2 contributed by leaf C content reflects conservative resources utilization. The direct negative effects of CWM_PC2_ on belowground biomass in the species richness experiment and on soil nutrient cycles in the genotype richness experiment could be explained by the mass ratio hypothesis, because species with higher conservative resources utilization can cause weaker soil nutrient cycling through rhizosphere effects (Henneron et al., 2020). However, the positive direct effect of CWM_PC2_ on aboveground biomass is contrary to the mass ratio hypothesis (Figure 2). In addition, CWM_PC2_ showed significant effects on biomass in the species richness experiment while on soil nutrient cycles in the genotype experiment, which demonstrates that the processes of the same ecosystem functions and mechanisms accordingly are different between the two richness systems at inter‐ and intraspecific levels.

## 5 CONCLUSION

The present study shows that the direct effects of species richness or genotype richness in the dominant species *S. grandis* on the soil P cycle are similar, which could be explained from the point of view of evolution, insurance hypothesis, and facilitative effects between plant and plant, considering the P deficiency in the semi‐arid temperate steppe of northern China. However, there are significant differences of the effects of community functional structure on ecosystem functions between the two richness experiments, which is related to the different resources utilization and biodiversity effects between inter‐ and intraspecific richness levels (Yang et al., 2021). These findings demonstrate the undeniably important effects of *S. grandis* genotype diversity on ecosystem functions in the semi‐arid steppe region and suggest the limitation of community functional structure in explaining the BEF relationships. The present study reveals the effects of biodiversity changes on ecosystem functioning from inter‐ and intraspecific perspectives, and provides a scientific basis for predicting community dynamics and ecosystem functioning in semi‐arid temperate grasslands.

## CONFLICT OF INTEREST

None declared.

## AUTHOR CONTRIBUTIONS

**Man Jiang:** Formal analysis (lead); writing–original draft (lead); writing–review and editing (equal). **Xue Yang:** Conceptualization (equal); data curation (equal). **Tao Wang:** Data curation (equal); writing–review and editing (equal). **Yujuan Xu:** Writing–review and editing (equal). **Ke Dong:** Software (equal). **Luoyang He:** Formal analysis (equal). **Yulin Liu:** Data curation (equal). **Jinlong Wang:** Funding acquisition (equal); methodology (equal). **Nianxi Zhao:** Funding acquisition (equal); writing–review and editing (equal). **Yubao Gao:** Methodology (equal).

## Supporting information

Appendix S1

## Data Availability

Data are accessible on Dryad (https://doi.org/10.5061/dryad.ncjsxksvw).
