# Supplementary material for: A direct comparison of the effects and mechanisms between species richness and genotype richness in a dominant species on multiple ecosystem functions
Source: Ecol Evol. 2021 Sep 16;11(20):14125–34. doi: 10.1002/ece3.8125 (PMC8525171; doi:10.1002/ece3.8125)
Supplement: Supplementary file 1 — Appendix S1 [file ECE3-11-14125-s001.docx]

**Supplementary methods and results**

**Belongs to the study:**

**A direct comparison of the effects and mechanisms between species richness and genotype richness of a dominant species on multiple ecosystem functions**

**Man Jiang, Xue Yang, Tao Wang, Yujuan Xu, Ke Dong, Luoyang He, Yulin Liu, Jinlong Wang, Nianxi Zhao, Yubao Gao**

**S1 Additional tables**

**Table S1** Dry mass of each species at the beginning of the experiments

| **Experiment** | **Species** | **Aboveground biomass dry weight (g/individual)** | **Belowground biomass dry weight**  **(g/individual)** | |
| --- | --- | --- | --- | --- |
| *Specie richness experiment* | *Achnatherum sibiricum* | 0.02004 | 0.01648 |  |
|  | *Agropyron Cristatum* | 0.01508 | 0.01272 |  |
|  | *Cleistogenes squarrosa* | 0.01518 | 0.0295 |  |
|  | *Leymus chinensis* | 0.02318 | 0.0252 |  |
|  | Poa pratensis | 0.01278 | 0.01868 |  |
|  | *Allium senescens* | 0.03582 | 0.02704 |  |
|  | *Potentilla bifurca* | 0.03908 | 0.02414 |  |
|  | *Potentilla acaulis* | 0.03038 | 0.02822 |  |
|  | *Serratula centauroides* | 0.1167 | 0.04094 |  |
|  | *Carex korshinskyi* | 0.02298 | 0.00718 |  |
|  | *Koeleria cristata* | 0.01252 | 0.01642 |  |
|  | *Artemisia frigida* | 0.01252 | 0.01642 |  |
| *Genotype richness experiment* | *Stipa grandis* | 0.01686 | 0.03228 |  |

**Table S2** Correlation coefficients between plant functional traits in the species richness experiment (below diagonal) and in the genotype richness experiment (above diagonal). The correlation coefficients higher than 0.7 are in bold style.

| Traits | Plant height | Plant width | Specific leaf area | Leaf dry matter content | Root volume | Leaf C content | Leaf N content | Leaf P content |
| --- | --- | --- | --- | --- | --- | --- | --- | --- |
| Plant height | **-** | 0.517 | -0.294 | -0.007 | 0.119 | 0.000 | -0.189 | -0.042 |
| Plant width | **0.790** | - | -0.252 | -0.462 | -0.154 | 0.441 | -0.252 | -0.021 |
| Specific leaf area | 0.622 | 0.587 | - | 0.650 | 0.287 | 0.287 | 0.329 | 0.245 |
| Leaf dry matter content | 0.462 | 0.098 | 0.490 | - | 0.601 | -0.294 | 0.028 | 0.042 |
| Root volume | 0.629 | 0.657 | 0.657 | 0.014 | - | -0.056 | -0.021 | 0.098 |
| Leaf C content | 0.622 | 0.322 | 0.343 | 0.517 | 0.252 | - | 0.441 | 0.483 |
| Leaf N content | 0.161 | 0.098 | 0.042 | -0.168 | 0.035 | 0.399 | - | **0.713** |
| Leaf P content | 0.098 | -0.231 | -0.196 | 0.266 | -0.448 | 0.497 | 0.350 | - |

**Table S3** Correlation coefficients between pair-wise soil variables about C, N, and P cycle in the species richness experiment (below diagonal) and in the genotype richness experiment (above diagonal).

| Variables | Soil total carbon content | Soil organic carbon content | β-glucosidase activity | Soil total nitrogen content | Ammonium (NH_4_^+^-N) | Nitrate (NO_3_^-^-N) | Nitrification rate | Mineralization rate | Soil urease activity | Available phosphate content | Phosphatase activity |
| --- | --- | --- | --- | --- | --- | --- | --- | --- | --- | --- | --- |
| Soil total carbon content | - | 0.229 | 0.013 |  |  |  |  |  |  |  |  |
| Soil organic carbon content | 0.220 | - | 0.019 |  |  |  |  |  |  |  |  |
| β-glucosidase activity | 0.172 | 0.114 | - |  |  |  |  |  |  |  |  |
| Soil total nitrogen content |  |  |  | - | 0.349 | -0.100 | -0.144 | 0.014 | -0.260 |  |  |
| Ammonium (NH_4_^+^-N) |  |  |  | 0.245 | - | -0.309 | -0.282 | 0.166 | -0.105 |  |  |
| Nitrate (NO_3_^-^-N) |  |  |  | 0.083 | 0.393 | - | 0.264 | -0.108 | -0.129 |  |  |
| Nitrification rate |  |  |  | -0.106 | 0.053 | 0.52 | - | -0.033 | -0.116 |  |  |
| Mineralization rate |  |  |  | -0.206 | -0.051 | -0.259 | -0.164 | - | 0.018 |  |  |
| Soil urease activity |  |  |  | 0.275 | 0.017 | -0.003 | -0.025 | 0.010 | - |  |  |
| Available phosphate content |  |  |  |  |  |  |  |  |  | - | 0.081 |
| Phosphatase activity |  |  |  |  |  |  |  |  |  | -0.292 | - |

**S2 Additional Figure**

**
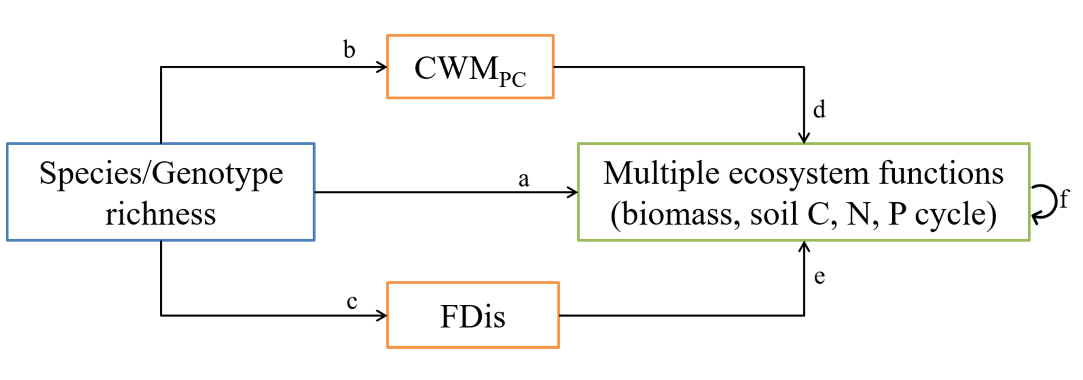
**

**Figure S1** *A prior* model based on the existing system theory.

The first level of the prior model is species richness or genotype richness; the second level is community functional structure: the community-weighted mean trait values (CWM_PC_) and function dispersion (FDis); the third level is multiple ecosystem functions (biomass, soil C, N, P cycle). a: Plant richness such as species richness or genotype species within a species can directly affect ecosystem functions (van der Plas et al., 2019). b, c, d, e: The plant community functional structure can mediate the responses of ecosystem functions to the changes of plant richness (Valencia et al., 2015; Xu et al., 2018) through the "mass ratio hypothesis" (Grime, 1998) and the niche complementarity hypothesis (Tilman, 1997; Díaz et al., 2007). f: Relationships between different ecosystem functions need to be considered, as ecosystem functions can simultaneously increase/decrease or trade off with each other along the plant richness gradient (Lefcheck, 2015; Wu et al., 2019).

**S3 References list**

Díaz, S., Lavorel, S., Bello, F.d., Quétier, F., Grigulis, K. & Robson, T.M. (2007). Incorporating plant functional diversity effects in ecosystem service assessments. *Proceedings of the National Academy of Sciences of the United States of America,* **104,** 20684-20689. <https://doi.org/10.1073/pnas.0704716104>

Grime, J.P. (1998). Benefits of plant diversity to ecosystems: immediate, filter and founder effects. *Journal of Ecology,* **86,** 902-910. <https://doi.org/10.1046/j.1365-2745.1998.00306.x>

Lefcheck, J.S. (2015). piecewiseSEM : Piecewise structural equation modelling in R for ecology, evolution, and systematics. *Methods in Ecology and Evolution,* **7,** 573-579. <https://doi.org/10.1111/2041-210x.12512>

Tilman, D., Knops, J., Wedin, D., Reich, P., Ritchie, M. & Siemann, E. (1997). The Influence of Functional Diversity and Composition on Ecosystem Processes. *Science,* **277,** 1300-1302. <https://doi.org/10.1126/science.277.5330.1300>

Valencia, E., Maestre, F.T., Bagousse‐Pinguet, Y.L., Quero, J.L., Tamme, R., Börger, L., García‐Gómez, M. & Gross, N. (2015). Functional diversity enhances the resistance of ecosystem multifunctionality to aridity in Mediterranean drylands. *New Phytologist,* **206,** 660-671. <https://doi.org/10.1111/nph.13268>

van der Plas, F. (2019). Biodiversity and ecosystem functioning in naturally assembled communities. *Biological Reviews,* **94,** 1220-1245. <https://doi.org/10.1111/brv.12499>

Xu, Z., Li, M., Zimmermann, N.E., Li, S., Li, H., Ren, H., Sun, H., Han, X., Jiang, Y. & Jiang, L. (2018). Plant functional diversity modulates global environmental change effects on grassland productivity. *Journal of Ecology*, **106**, 1941-1951. <https://doi.org/10.1111/1365-2745.12951>

Wu, H., Xiang, W., Ouyang, S., Forrester, D.I., Zhou, B., Chen, L., Ge, T., Lei, P., Chen, L., Zeng, Y., Song, X., Peñuelas, J. & Peng, C. (2019). Linkage between tree species richness and soil microbial diversity improves phosphorus bioavailability. *Functional Ecology,* **33,** 1549-1560. <https://doi.org/10.1111/1365-2435.13355>
